# The impact of phased university reopenings on mitigating the spread of COVID-19: a modeling study

**DOI:** 10.1186/s12889-021-11525-x

**Published:** 2021-08-06

**Authors:** Lior Rennert, Corey A. Kalbaugh, Christopher McMahan, Lu Shi, Christopher C. Colenda

**Affiliations:** 1 Department of Public Health Sciences, Clemson University, 529 Edwards Hall, Clemson, SC USA; 2 School of Mathematical and Statistical Sciences, Clemson University, Clemson, SC USA; 3 Department of Internal Medicine, Section of Gerontology and Geriatrics, Wake Forest University, Winston-Salem, NC USA

**Keywords:** COVID-19, SARS-CoV-2, Testing, Mitigation, Students, University, Phased return, Modeling

## Abstract

**Background:**

Several American universities have experienced COVID-19 outbreaks, risking the health of their students, employees, and local communities. Such large outbreaks have drained university resources and forced several institutions to shift to remote learning and send students home, further contributing to community disease spread. Many of these outbreaks can be attributed to the large numbers of active infections returning to campus, alongside high-density social events that typically take place at the semester start. In the absence of effective mitigation measures (e.g., high-frequency testing), a phased return of students to campus is a practical intervention to minimize the student population size and density early in the semester, reduce outbreaks, preserve institutional resources, and ultimately help mitigate disease spread in communities.

**Methods:**

We develop dynamic compartmental SARS-CoV-2 transmission models to assess the impact of a phased reopening, in conjunction with pre-arrival testing, on minimizing on-campus outbreaks and preserving university resources (measured by isolation bed capacity). We assumed an on-campus population of *N* = 7500, 40% of infected students require isolation, 10 day isolation period, pre-arrival testing removes 90% of incoming infections, and that phased reopening returns one-third of the student population to campus each month. We vary the disease reproductive number (*R*_*t*_) between 1.5 and 3.5 to represent the effectiveness of alternative mitigation strategies throughout the semester.

**Results:**

Compared to pre-arrival testing only or neither intervention, phased reopening with pre-arrival testing reduced peak active infections by 3 and 22% (*R*_*t*_ = 1.5), 22 and 29% (*R*_*t*_ = 2.5), 41 and 45% (*R*_*t*_ = 3.5), and 54 and 58% (improving *R*_*t*_), respectively. Required isolation bed capacity decreased between 20 and 57% for values of *R*_*t*_ ≥ 2.5.

**Conclusion:**

Unless highly effective mitigation measures are in place, a reopening with pre-arrival testing substantially reduces peak number of active infections throughout the semester and preserves university resources compared to the simultaneous return of all students to campus. Phased reopenings allow institutions to ensure sufficient resources are in place, improve disease mitigation strategies, or if needed, preemptively move online before the return of additional students to campus, thus preventing unnecessary harm to students, institutional faculty and staff, and local communities.

**Supplementary Information:**

The online version contains supplementary material available at 10.1186/s12889-021-11525-x.

## Background

Higher education institutions are struggling to reopen their campuses in a safe and judicious manner during the Coronavirus disease 2019 (COVID-19) pandemic. The reopening strategies for Fall 2020 implemented by several major universities in the United States have been largely unsuccessful [1]. Other institutions have elected to delay reopening, partially reopen, or remain closed, preferring instead to continue with online instruction [2–4]. Those planning to continue with reopening are exploring several preventative strategies to mitigate the spread of severe acute respiratory syndrome coronavirus 2 (SARS-CoV-2), including frequent testing, contact tracing, and isolation of confirmed and suspected cases [5, 6].

Controlling campus outbreaks is essential for both student and community health. As of March 2nd, 2020, American colleges and universities have reported more than 535,000 cases since reopening [7]. While this population has reported a relatively low mortality rate compared to the general population, such large outbreaks will lead to a large number of symptomatic students who may be at substantial risk for post-acute COVID [8]. Outbreaks on campus will also inevitably lead to a mass increase in infections among faculty, staff, and local communities [1]. A recent modeling study found that reopening American college and university campuses may lead to an additional 820 community infections for every 10,000 residents throughout the semester [9].

One of the first steps to preventing large outbreaks is minimizing the number of infectious students returning to campus. Previous modeling studies have demonstrated that high numbers of active infections at the beginning of the semester lead to early and large outbreaks and drain institutional resources [10]. This has been further evidenced by recent COVID-19 outbreaks in major universities, [1] which have been forced to send students home and shift to online learning within 1 week of reopening [11, 12]. With such early outbreaks and closures, implementation of preventative strategies throughout the semester may no longer be relevant, as these strategies are intended to prevent outbreaks rather than contain them. Indeed, the initial number of active infections assumed by modeling studies that support these strategies [13, 14] may be far lower than suggested by current estimates of SARS-CoV-2 prevalence and recent university reopenings [11, 12]. Contact tracing, for example, has shown to be ineffective when the number of initial infections is greater than 40 [13]. Colleges and universities intending to reopen campuses in future semesters must therefore place a greater emphasis on reducing active infections and limiting outbreaks at the semester start [1]. However, between the large number of students who live in congregate housing [1] and the number of high-density social gatherings that occur early in the semester and beyond, [11, 15, 16] such outbreaks may be difficult to contain with the simultaneous return of all students to campus [17]. While evidence shows that high-frequency repeated testing is effective in mitigating disease spread on college and university campuses, [5, 18] many institutions lack the testing capacity necessary for implementation [19]. In the absence of such testing, alternative strategies for disease mitigation are needed.

A phased return of students to campus is a practical intervention to limit early outbreaks, ensure proper protocols are in place, and preserve university resources. This is accomplished through minimizing the susceptible population size and density early in the semester, which can delay large outbreaks and reduce outbreak size, ensure the availability of sufficient resources by vacating a large portion of isolation beds for confirmed or suspected cases, and increase testing and support service capacity per student. Furthermore, a phased reopening provides institutions time to improve strategies to mitigate the spread of COVID-19 (e.g., frequent testing [1]) and adjust for factors that drive outbreaks (e.g., fraternity gatherings [11, 15, 16]) before the return of additional students to campus. If outbreaks cannot be contained, a phased reopening allows higher education institutions to pre-emptively transition to remote learning before the arrival of all students and thus prevent unneccessary harm to students, faculty and staff, and local communities.

Our team was tasked with recommending strategies to limit outbreaks and ensure adequate resources are in place for confirmed COVID-19 cases in a large university in the Southeastern United States during the Fall 2020 semester. To guide and inform our recommendations, we developed dynamic compartmental transmission models to assess the impact of a phased reopening, along with exclusion of COVID-19 positive students through testing prior to campus arrival, on minimizing outbreak size and preserving university resources throughout the semester. Preservation of university resources is important to ensure adequate student care and limit community spread. Without sufficient resources, such as isolation beds, universities may be forced to send students home which may increase disease spread in their home communities [20].

## Methods

**Fig. 1:**
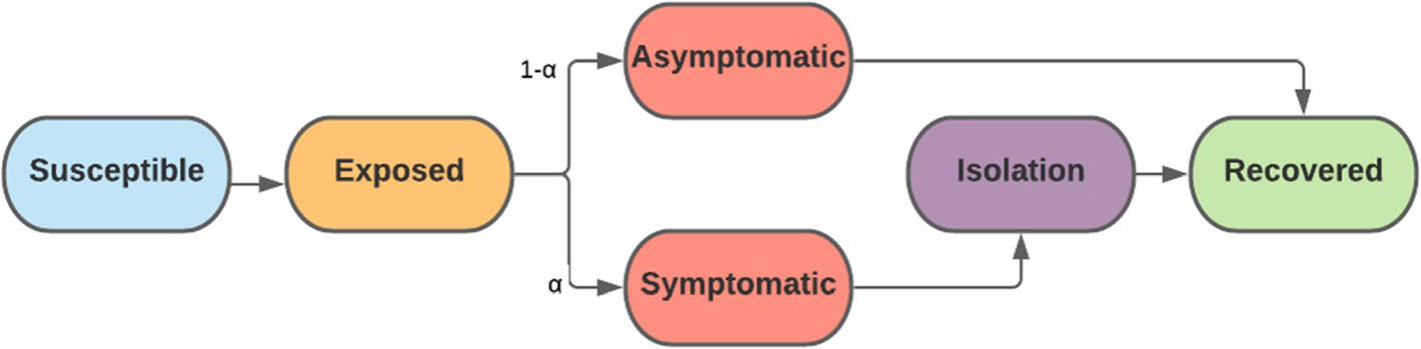
Model structure. The population is divided into the following six compartments: susceptible, exposed (not infectious or detectable), asymptomatic (infectious and not detectable), symptomatic (infectious and detectable), isolated (no contact with other individuals), and recovered. Exposed individuals transition into the symptomatic compartment with probability α and asymptomatic compartment with probability 1-α. It is assumed all symptomatic individuals are detected (after an average period of 3 days) and isolated for the remaining duration of their infection

To capture the essential features of COVID-19 spread on campus, we developed dynamic compartmental transmission models of SARS-COV-2 [21] with the following compartments: susceptible, exposed, infectious.

(asymptomatic/undetected), infectious (symptomatic/detected), isolated, and recovered (Fig. 1). We assumed a large on-campus population (*N* = 7500), an active infection rate of 3% at the semester start, [18] and that 40% of active infections would be detected [18] and require isolation for an average period of 10 days [22]. We considered four settings for the reproductive number (*R*_*t*_) to represent the effectiveness of various mitigation strategies throughout the semester: highly effective (*R*_*t*_ = 1.5), moderately effective (*R*_*t*_ = 2.5), and ineffective (*R*_*t*_ = 3.5), [5] along with a time-varying *R*_*t*_ that improved throughout the semester (*R*_*0*_ = 3.5, *R*_*1*_ = 2.5, and *R*_*t*_ = 1.5 for months *t* ≥ 2). In the former setting, we assume that *R*_*t*_ = *R*_0_ for all *t* ≥ 0. The latter setting was intended to capture improvement in mitigation efforts over time, such as an increase in testing capabilities or greater enforcement of mask mandates. Additional assumptions were compiled from published sources and are provided in Table 1. Because this study used only a theoretical model with no human subject data, the Institutional Review Board of Clemson University determined that this research did not involve human participants and did not require their approval.

**Table 1:** Model input parameters, assumptions, and references

Model parameter	Input
On-campus population (N)	7500 (assumption)
Time horizon (weeks)	18 weeks
Disease dynamics ^a^	
Mean incubation time, 1/σ	3 days [23]
Mean asymptomatic infectious time (days), 1/φ	10 days [22]
Mean symptomatic infection time before detection and isolation (days), 1/γ	3 days (accounting for 2-day pre-symptomatic period and 1-day test turnaround time) [23]
Isolation time, 1/ρ (days)	10 days [22]
Proportion of infections that are symptomatic, α	0.4 [18]
Transmission rate, β	Dependent on *R*_*t*_
Baseline infectious rate (%) ^b^	3% [18]
Baseline recovered rate (%)	10% [10, 14, 24]
**Mitigation strategies throughout semester (*R***_***t***_**)** ^c^	
Highly effective (best case)	1.5 [5]
Moderately effective (base case)	2.5 [5]
Ineffective (worst case)	3.5 [5]
Time-varying	Month 0: 4; Month 1: 2.5; Month 2+: 1.25 (assumption)
**Interventions**	
Test characteristics	
Sensitivity (%)	90% [5, 25]
Specificity (%)	100% (assumption)
Phased re-opening	
Phase 1: Calendar time (months)/sub-population returning to campus	0 months/2500 students (assumption)
Phase 2: Calendar time (months)/sub-population returning to campus/cumulative population	1 months/2500 students/5000 students (assumption)
Phase 3: Calendar time (months)/sub-population returning to campus/cumulative population	2 months/2500 students/7500 students (assumption)

^a^ We assume a closed system (i.e., no exogenous infections or deaths)

^b^ Under pre-arrival testing, baseline infections are reduced by 90%

^c^ Under phased reopening, we assume this number is reduced by 20% during the first phase and 10% during the second phase

We considered three interventions: Phased reopening with pre-arrival testing, pre-arrival testing only, and neither intervention. We assumed that pre-arrival testing reduced the number incoming infections by 90% [5, 25]. To reduce model complexity, COVID-19 positive individuals detected through pre-arrival testing immediately entered the isolation compartment and undetected infections entered the asymptomatic and symptomatic infectious compartments (according to α parameter in Table 1). We assumed a phased re-opening over a 2-month period, in which one-third of the population (2500 students) returned to campus at the semester start, 30 days after the semester start, and 60 days after the semester start. We further assumed that the disease reproductive rate is reduced by 20% during the first phase and 10% during the second phase due to a decrease in student population density, and that any unoccupied beds during these phases are available for isolation of detected COVID-19 cases. The baseline infection rate for incoming students was held constant at 3% in all phases. The equations and initial values for each compartment are provided in Supplementary Table 1.

We evaluated the relative impact of each intervention on the number of active infections throughout the semester (daily, peak, total) and isolation bed capacity. At each timepoint *t*, active infections was defined as the number of currently infected students (i.e., sum of asymptomatic, symptomatic, and isolation compartments at time *t*). Peak active infections was defined as the maximum number of daily active infections. Isolation bed capacity was measured as the number of on-campus beds needed for isolation throughout the semester (*n*_*beds*_) and as the proportion relative to the on-campus population size (*n*_*beds*_/*N*). In the Supplementary Analyses, we explore the relative impact of a phased reopening on infections and isolation bed capacity under several scenarios: 1) Higher proportion of asymptomatic individuals, 2) decreased test sensitivity at the semester start, 3) larger student immunity at the semester start, 4) shorter time periods between phases and 5) faster implementation of effective mitigation measures. A publicly accessible version of the model implementation is available online (https://rennertl.shinyapps.io/phasedreopeningprojections).

## Results

**Fig. 2:**
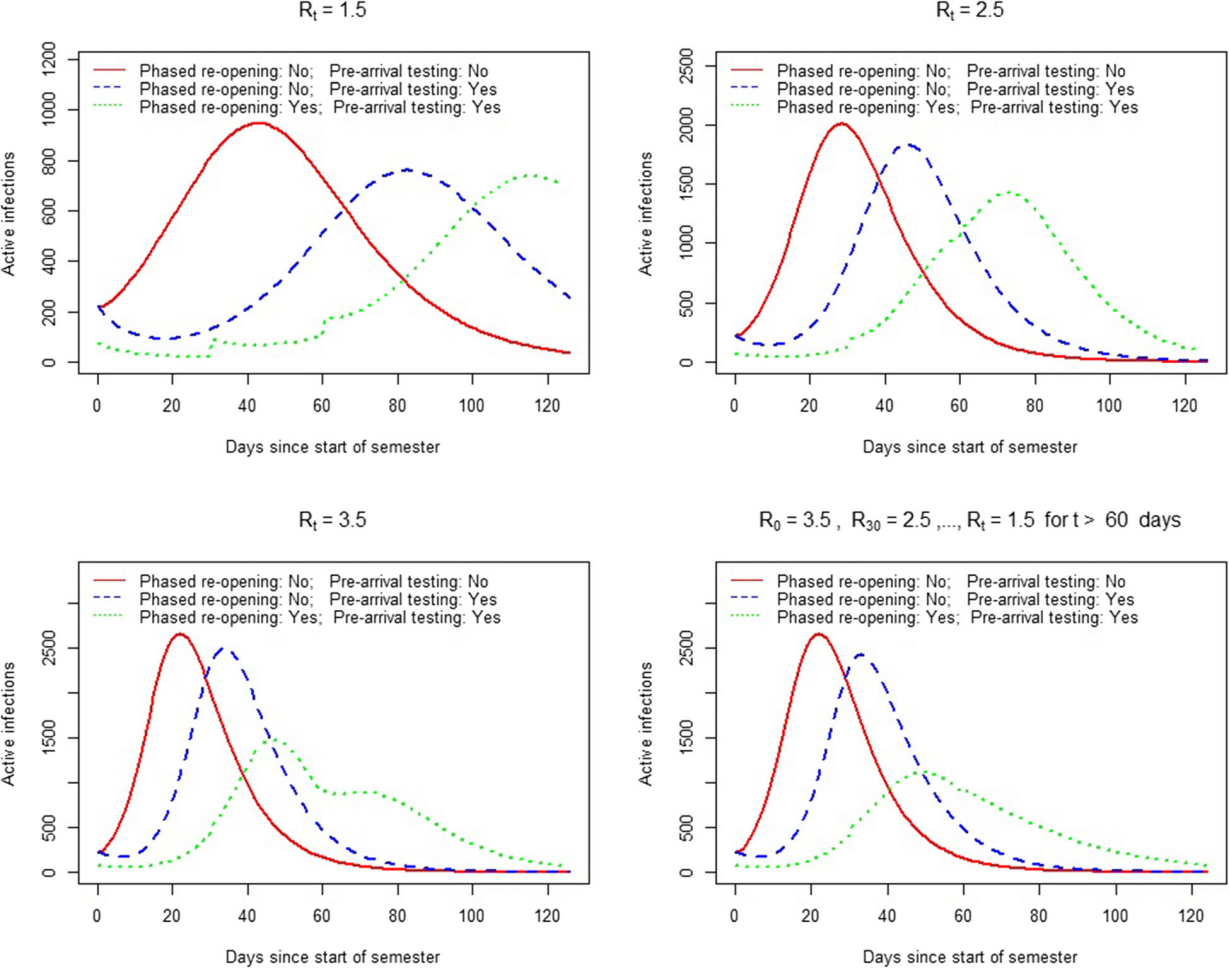
Projected active infections (daily) throughout the semester for each intervention**.** Expected number of active infections throughout the semester under three interventions: No phased reopening or pre-arrival testing (solid red line), no phased reopening with pre-arrival testing (dashed blue line), phased reopening with pre-arrival testing (dotted green line). Top left panel: effective mitigation strategies throughout semester (*R*_*t*_ = 1.5). Top right panel: moderately effective mitigation strategies throughout the semester (*R*_*t*_ = 2.5). Bottom left panel: ineffective mitigation strategies throughout the semester (*R*_*t*_ = 3.5). Bottom right panel: improving mitigation strategies throughout the semester (*R*_*0*_ = 3.5, *R*_*1*_ = 2.5, and *R*_*t*_ = 1.5 for months *t* ≥ 2)

The number of active infections throughout the semester based on the model projections are displayed in Fig. 2. In all scenarios, both pre-arrival testing alone and in conjunction with phased reopening reduced the rate of active infections early in the semester and delayed the timing of the peak outbreak size. Summary statistics are displayed in Table 2. A phased reopening in conjunction with pre-arrival testing reduced the size of the peak outbreak across all scenarios. Under highly effective mitigation strategies throughout the semester (*R*_*t*_ = 1.5), a phased reopening reduced peak outbreak size by 3 and 22% compared to pre-arrival testing only and no interventions, respectively. For moderate to ineffective mitigation strategies throughout the semester, this decrease was substantial. Compared to the simultaneous return of all students, a phased reopening (with pre-arrival testing) decreased the peak number of active infections between 22 to 29% (*R*_*t*_ = 2.5) and 41 to 45% (*R*_*t*_ = 3.5). Under improving mitigation strategies throughout the semester (*R*_*0*_ = 3.5, *R*_*1*_ = 2.5, and *R*_*t*_ = 1.5 for *t* ≥ 2 months), phased reopening with pre-arrival testing decreased the peak number of active infections between 54 to 58%. Under effective (*R*_*t*_ = 1.5) and improving mitigation strategies, a phased return of students to campus reduced total infections by 22 and 28% throughout the semester. The relative decrease under moderate (*R*_*t*_ = 2.5) and infective (*R*_*t*_ = 3.5) mitigation measures was between 2 and 5%. Compared to no interventions, pre-arrival testing alone reduced peak active infections between 6 and 20% and reduced total infections between 0.2 and 8% across all scenarios.

**Table 2:** Outcome metrics based on modeling study under each strategy across varying disease reproductive numbers

Phased reopening	Pre-arrival testing	Peak outbreak: size (days to peak)			
		***R***_***t***_ **= 1.5**	***R***_***t***_ **= 2.5**	***R***_***t***_ **= 3.5**	***R***_***t***_ **= 3.5, 2.5, 1.5** ^a^
No	No	948 (42 *days*)	2014 (28 *days*)	2660 (22 *days*)	2660 (22 *days*)
No	Yes	760 (82 *days*)	1837 (46 *days*)	2495 (34 *days*)	2422 (33 *days*)
Yes	Yes	738 (115 *days*)	1430 (73 *days*)	1476 (47 *days*) ^b^	1111 (49 *days*) ^b^
		**Total infections**			
		***R***_***t***_ **= 1.5**	***R***_***t***_ **= 2.5**	***R***_***t***_ **= 3.5**	***R***_***t***_ **= 3.5, 2.5, 1.5** ^a^
No	No	4974	6192	6438	6394
No	Yes	4562	6151	6425	6261
Yes	Yes	3558	6010	6100	4856
		**Beds needed for isolation of detected individuals:** ***n***_***beds***_ **(%)** ^**c**^			
		***R***_***t***_ **= 1.5**	***R***_***t***_ **= 2.5**	***R***_***t***_ **= 3.5**	***R***_***t***_ **= 3.5, 2.5, 1.5** ^a^
No	No	336 (4.5%)	704 (9.4%)	916 (12.2%)	916 (12.2%)
No	Yes	270 (3.6%)	643 (8.6%)	863 (11.5%)	834 (11.1%)
Yes	Yes	263 (3.5%)	513 (6.8%)	415 (5.5%)	397 (5.3%)

Outcome metrics are peak outbreak size (and days to peak outbreak size), total infections, and isolation beds capacity for detected students throughout the semester under three interventions: No phased reopening or pre-arrival testing, pre-arrival testing only, phased re-opening with pre-arrival testing. The size of the on-campus student population is *N* = 7500

^*a*^ Improving *R*_*t*_: *R*_*0*_ = 3.5, *R*_*1*_ = 2.5, and *R*_*t*_ = 1.5 for months *t* ≥ 2

^*b*^ Peak outbreak occurred with 2/3’s of student population on campus (i.e, 5000 students)^c^ Proportion of isolation beds needed (*n*_*beds*_) relative to on-campus student population (*N*)

Pre-arrival testing alone had minimal impact on maximum isolation bed capacity, while implementation in conjunction with a phased reopening substantially reduced the number of isolation beds needed for values of *R*_*t*_ ≥ 2.5 (Table 2). Under *R*_*t*_ = 2.5, phased reopening with pre-arrival testing required 6.8% of beds reserved for isolation of symptomatic/detected students, a 20 to 27% reduction compared to pre-arrival testing only and no interventions, respectively. Under *R*_*t*_ = 3.5 and improving *R*_*t*_, phased reopening in conjunction with pre-arrival testing required 5.3 to 5.5% of beds reserved for isolation of symptomatic students - a 52 to 57% decrease compared to the two other strategies. .

## Discussion

Minimizing the number of active infections at the semester start is essential to limiting rapid outbreaks and ensuring sufficient resources are available for support services such as testing, contact tracing, and case isolation. Universities implementing frequent SARS-CoV-2 testing have been relatively successful in detecting and containing outbreaks [18, 26]. However, most universities did not regularly test their students during the Fall 2020 semester [19]. Our study found that unless highly effective mitigation strategies are implemented, such as frequent testing, the simultaneous arrival of all students to campus leads to early and large outbreaks. Furthermore, the timing of these outbreaks occur early in the semester and are therefore not impacted by improvements in mitigation efforts over time. Our modeling study concluded that a phased return of students to campus in conjunction with testing prior to arrival substantially delays the peak outbreak timing and reduces the outbreak size by up to 58%.

Rapid outbreaks drain university resources and have lead to institutions shutting down on-campus activities and shifting fully online [1]. Our models demonstrate that the simultaneous return of all students to campus may require reservation of over 10% of on-campus beds for isolation of detected COVID-19 cases, far exceeding the current capacity of several large institutions [27, 28]. In fact, we found that the recommended 5% of isolation beds for confirmed COVID-19 cases [29] may lead to institutions reaching capacity in less than 2 weeks. Under a phased reopening with pre-arrival testing, the number of isolation beds needed ranged from 3.5 to 6.8% of total capacity. A phased return would further decrease the number of beds needed if students testing positive prior to arrival stayed at home rather than occupying a university-provided isolation bed.

Phased reopenings are practical interventions that can be implemented in various ways. During the Fall 2020 semester, Clemson University phased the return of students to campus by beginning the semester entirely online and delaying in-person instruction by 1 month [18]. The phased return of off-campus students did not require explicit interventions since these students (roughly two-thirds of the undergraduate population) were either already living off campus at the start of summer 2020, or gradually moved off-campus throughout the summer months as new leases were enacted. However, delaying in-person instruction and access to residential buildings by 1 month ensured that residential and non-residential students would not simultaneously arrive to campus or the immediate off-campus area. Furthermore, the return of residential students to campus was distributed over a 10-day period to further decrease the risk of outbreaks. We note that the phased return of students to campus was only applied during the Fall semester when a large portion of the population was susceptible to infection. As more of the population builds immunity through natural infection, [30] the relative impact of a phased a student return on disease mitigation is less substantial.

Based on mandatory pre-arrival and survellance testing throughout the Fall 2020 semester, there is evidence that infections among off-campus students reached their peak during the summer months of 2020 and thus prior to in-person instruction [18]. On the other hand, peak infection among on-campus students occurred in during in-person instruction of the fall semester [18]. Approximately 620 of the 800 reserved isolation beds were in use following the peak infection period. If both on- and off-campus populations had returned to the campus area simultaneously, it is possible that peak infection would have been greater among on-campus students (due to increased transmission from off-campus students) and may have forced the university to suspend or shut down operations for the remainder of the semester.

Our study has several limitations. First, we omit an in-depth discussion of the logistics behind a phased reopening. Difficulties in implementation include the careful coordination of the return of students back to campus and may require institutions to shift between on-campus and remote learning throughout the phased reopening. The costs of unutilized institutional facilities are also not considered here. In addition, our study does not consider the contribution of off-campus students to the spread of COVID-19, and we do not explicitly model the impact of a phased reopening on community spread. As empirical data becomes available, future research must examine the impact of university outbreaks in local communities along with the impact of university-level interventions on mitigating disease spread in these communities.

Another limitation is that we provide estimates for isolation bed capacity of detected students only. The total number of beds needed to be reserved by institutions must also account for quarantining close contacts of detected positive individuals [31]. Therefore, the numbers presented here are likely underestimating the true number of reserved beds needed. Furthermore, if institutions implement surveillance testing, additional beds would be needed for asymptomatic cases. However, the total number of required beds may ultimately be lower if frequent testing was employed [5].

While we used current evidence to inform plausible biological parameters for SARS-CoV-2 transmission, these values may need refinement as more data become available (e.g., differing transmission rates between asymptomatic and symptomatic individuals) [32]. To assess sensitivity to model assumptions, we varied disease transmission parameters in order to provide a range of possible outcomes. While the effects of mitigation strategies throughout the semester, such as frequent testing, successful contact tracing, and quarantine of suspected cases are implicitly incorporated into our model through the reproductive number (*R*_*t*_), we do not consider the impact of these strategies on isolation bed occupancy. Finally, this study only considers a single phased reopening strategy (i.e., monthly return of one-third of student population). We have therefore created a free web-based application to allow for the exploration of alternative strategies under varying parameter values (available at: https://rennertl.shinyapps.io/phasedreopeningprojections/).

## Conclusions

As colleges and universities across the United States plan a return to normal campus life in the Fall 2021 semester, [33] we encourge institutions to ensure a sufficient supply of isolation beds and support service capacity. This is necessary to guarantee adequate student care and limit downstream effects on communities across the country. In addition, all students should be tested prior to campus arrival to avoid the return of active infections to campus. A phased reopening offers several additional benefits. Limiting the number of students on-campus at the start of the semester can substantially reduce the number of initial infections and delay the timing and size of outbreaks, while providing opportunities to improve safety protocols and adjust for factors that drive these outbreaks before the return of additional students to campus. Furthermore, minimizing the size of the susceptible population will help ensure that institutions have sufficient resources at their disposal to handle early outbreaks. A phased reopening also provides opportunities to trial mitigation strategies on a smaller population before they are implemented in larger scales. Most importantly, if COVID-19 outbreaks cannot be kept under control with a limited student population, phased reopenings provide the ability to halt the return of additional students to campus and prevent thousands of additional infections, thus preventing unnecessary harm to students, institutional faculty and staff, and local communities.

## Supplementary Information

**Additional file 1: Supplementary Table 1.** Equations and initial values for dynamic compartmental transmission models.

**Additional file 2: Supplementary Fig. 1.** Increasing the proportion of asymptomatic students to 75%. **Supplementary Fig. 2.** Decreasing pre-arrival test sensitivity to 70%. **Supplementary Fig. 3.** Increasing proportion of immune individuals at the semester start to 25%. **Supplementary Fig. 4.** Decreasing time between phases to 10 days. **Supplementary Fig. 5.** Improving *R*_*t*_ under settings in Supplementary Fig. 4.

## Data Availability

No data was collected for this study. A publicly accessible version of the model implementation is available online (https://rennertl.shinyapps.io/phasedreopeningprojections). Data sources that support our choice for model parameters are provided in Table 1.

## Abbreviations

Covid-19: Coronavirus disease 2019
SARS-CoV-2: Severe acute respiratory syndrome coronavirus 2
