# Supplementary material for: The impact of phased university reopenings on mitigating the spread of COVID-19: a modeling study
Source: BMC Public Health. 2021 Aug 6;21:1520. doi: 10.1186/s12889-021-11525-x (PMC8343346; doi:10.1186/s12889-021-11525-x)
Supplement: Supplementary file 1 — Additional file 1: Supplementary Table 1. Equations and initial values for dynamic compartmental transmission models. [file 12889_2021_11525_MOESM1_ESM.docx]

**Supplementary Table 1: Equations and initial values for dynamic compartmental transmission models**

| **Compartment** | **Equation** | **Initial parameters for each strategy** | | | | |
| --- | --- | --- | --- | --- | --- | --- |
|  |  | **No action** | **Pre-arrival testing** | **Phased reopening  with pre-arrival testing** | | |
|  |  | Day 0 ^a^ | Day 0 ^a^ | Day 0 ^b^ | Day 30 ^c^ | Day 60 ^a^ |
| Susceptible (S) | $\mathrm{dS}\left( t \right)=-\beta\times\left( A+I \right)\times S/N$ | S_0_ = 6525 | S_0_ = 6525 | S_0_ = 2175 | S_30_ + S_0_ | S_60_ + S_0_ |
| Exposed (E) | $\mathrm{dE}\left( t \right)= \beta\times\left( A+I \right)\times S/N- \sigma\times E$ | E_0_ = 0 | E_0_ = 0 | E_0_ = 0 | E_30_ + E_0_ | E_60_ + E_0_ |
| Asymptomatic (A) | $\mathrm{dA}\left( t \right)= \left( 1-\alpha\right)\times\sigma\times E$ –$\phi\times A$ | A_0_ = 135 | A_0_ = 13.5 | A_0_ = 4.5 | A_30_ + A_0_ | A_60_ + A_0_ |
| Symptomatic (I) | $\mathrm{dI}\left( t \right)= \alpha\times\sigma\times E$ - $\gamma\times I$ | I_0_ = 90 | I_0_ = 9 | I_0_ = 3 | I_30_ + I_0_ | I_60_ + I_0_ |
| Isolation (Q) | $\mathrm{dQ}\left( t \right)= \gamma\times I$ - $\rho\times Q$ | Q_0_ = 0 | Q_0_ = 202.5 | Q_0_ = 67.5 | Q_30_ + Q_0_ | Q_60_ + Q_0_ |
| Recovered (R) | $\mathrm{dR}\left( t \right)= \phi\times A$ + $\rho\times Q$ | R_0_ = 750 | R_0_ = 750 | R_0_ = 250 | R_30_ + R_0_ | R_60_ + R_0_ |

Models assume population size of *N* = 7500 and 3% active infection rate at semester start (day 0). S_j_, E_j_, A_j_, I_j_, Q_j_, and R_j_ indicate the number of individuals in each compartment on day *j* (e.g., A_0_ is the number of asytompatic individuals on day 0). Infectious individuals return to campus through compartment A and I. When no action is taken, the number of asymptomatic and symptomatic infected individuals on day 0 is given by A_0_ = *N* • 0.03∙(1-α) and I_0_ = *N* • 0.03∙α, where α is the proportion of infections that are symptomatic and is set to 0.4 in the main analysis. Pre-arrival testing assuming 90% test sensitivity yields A_0_ = *N* • 0.03∙(1-α) • 0.90 and I_0_ = *N* • 0.03∙α • 0.90.

^a^ Sum of compartments: 7500

^b^ Sum of compartments: 2500

^c^ Sum of compartments: 5000
