# Supplementary material for: The impact of phased university reopenings on mitigating the spread of COVID-19: a modeling study
Source: BMC Public Health. 2021 Aug 6;21:1520. doi: 10.1186/s12889-021-11525-x (PMC8343346; doi:10.1186/s12889-021-11525-x)
Supplement: Supplementary file 2 — Additional file 2: Supplementary Fig. 1. Increasing the proportion of asymptomatic students to 75%. Supplementary Fig. 2. Decreasing pre-arrival test sensitivity to 70%. Supplementary Fig. 3. Increasing proportion of immune individuals at the semester start to 25%. Supplementary Fig. 4. Decreasing time between phases to 10 days. Supplementary Fig. 5. Improving Rt under settings in Supplementary Fig. 4. [file 12889_2021_11525_MOESM2_ESM.docx]

**Supplementary Analyses**

We explore the relative impact of a phased reopening on infections and isolation bed capacity under several scenarios: 1) Higher proportion of asymptomatic individuals, 2) decreased test sensitivity at the semester start, 3) larger student immunity at the semester start, 4) shorter time periods between phases and 5) faster implementation of effective mitigation measures. Unless explicitly stated otherwise, input parameters in the following sensitivity analysis are set to the values provided in Table 1 of the main paper.

**Scenario 1. Increasing proportion of asymptomatic individuals.** In Supplementary Figure 1, we increase the proportion of asymptomatic individuals 75% under two scenarios: *R_t_* = 2.5 and improving *R_t_* (3.5 to 1.5 over 60 day period). Doing so increases total infections throughout the semester, but decreases the number of reserved isolation beds for symptomatic students.

**Scenario 2. Decreasing pre-arrival test sensitivity.** In Supplementary Figure 2, we decrease test sensitivity to 70% under two scenarios: *R_t_* = 2.5 and improving *R_t_* (3.5 to 1.5 over 60 day period). This leads to an increase in peak infection size and isolation bed capacity under pre-arrival testing. However, this also results in a shorter time period between the semester start and peak infection. Therefore, under a phased return of students to campus, more students are infected by the conclusion of the first phase. This results in less infections on campus that can interact with incoming students from later phases and therefore decreases peak infection size and isolation bed capacity.

**Scenario 3. Increasing student immunity at the semester start.** In Supplementary Figure 3, we increase student immunity from 10% to 25% by increasing the proportion of students who are recovered at the semester start under two scenarios: *R_t_* = 2.5 and improving *R_t_* (3.5 to 1.5 over 60 day period). For institutions reopening in the Fall 2020 semester, this scenario is representative of initial conditions for institutions returning students in the Spring 2021 semester.^1^ Disease transmission is decreased across all scenarios due to a smaller susceptible population and a decreased interaction rate between susceptible and infections individuals. This results in a decrease in peak outbreak size and isolation bed capacity. Under a base rate of *R_t_* = 2.5, the relative benefits of a phased reopening is minimized compared to the other strategies. However, under high transmission rates at the semester start (i.e., *R_t_* = 3.5 for *t* = 0 to 29 days), the benefits of a phased reopening are still substantial due to early and large outbreaks under strategies involving the simultaneous return of all students to campus in this scenario.

**Scenario 4. Shorter time periods between phases.** In Supplementary Figure 4, we decrease the time between phases from 30 days to 10 days under 2 scenarios: *R_t_* = 2.5 and *R_t_* = 3.5. Under the phased reopening, the total number of infections, peak outbreak size, and number of isolation beds needed all increase. In this scenario, there is little difference between a phased reopening with pre-arrival testing and pre-arrival testing only.

**Scenario 5. Faster implementation of effective mitigation measures** We repeat the analyses in scenario 4, this time under improving mitigation *R_t_* (3.5 to 1.5 over a 20-day period and 3.5 to 0.8 over a 20-day period). The results are presented in Supplementary Figure 5. The total number of infections drop compared to scenario 4, with a substantial decrease in peak outbreak size and isolation bed capacity for strategies involving pre-arrival testing. Under strong improvement in mitigation strategies (*R_t_* ranges from 3.5 to 0.8 over 20 days), there is a substantial benefit to a phased reopening with shorter time periods in between. In this situation, a phased reopening reduces isolation bed capacity by 39% and total infections by 60% throughout the semester.

**References**

1. Rennert L, McMahan C, Kalbaugh CA, et al. Surveillance-based informative testing for detection and containment of SARS-CoV-2 outbreaks on a public university campus: an observational and modelling study. *The Lancet Child & Adolescent Health*. Published online March 19, 2021. doi:10.1016/S2352-4642(21)00060-2


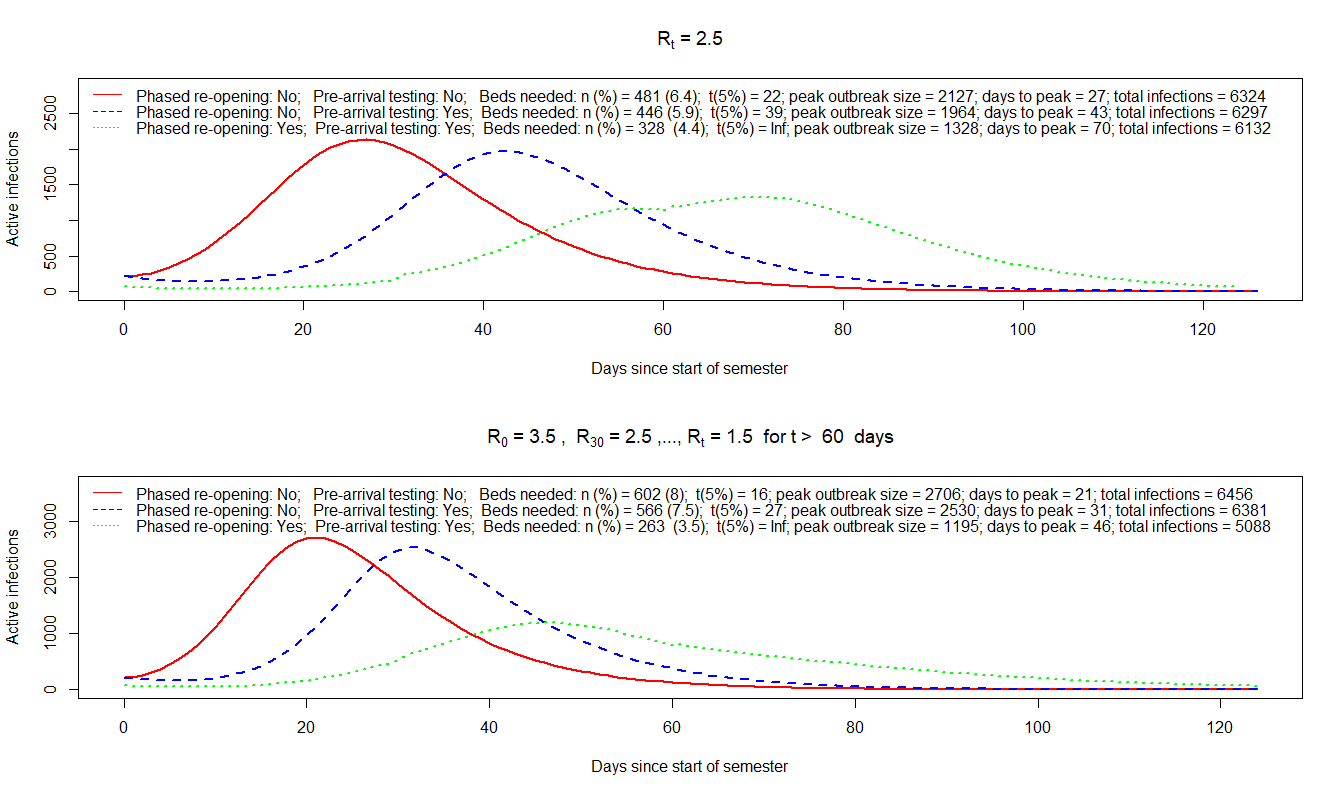


**Supplementary Figure 1.** Increasing the proportion of asymptomatic students to 75%.


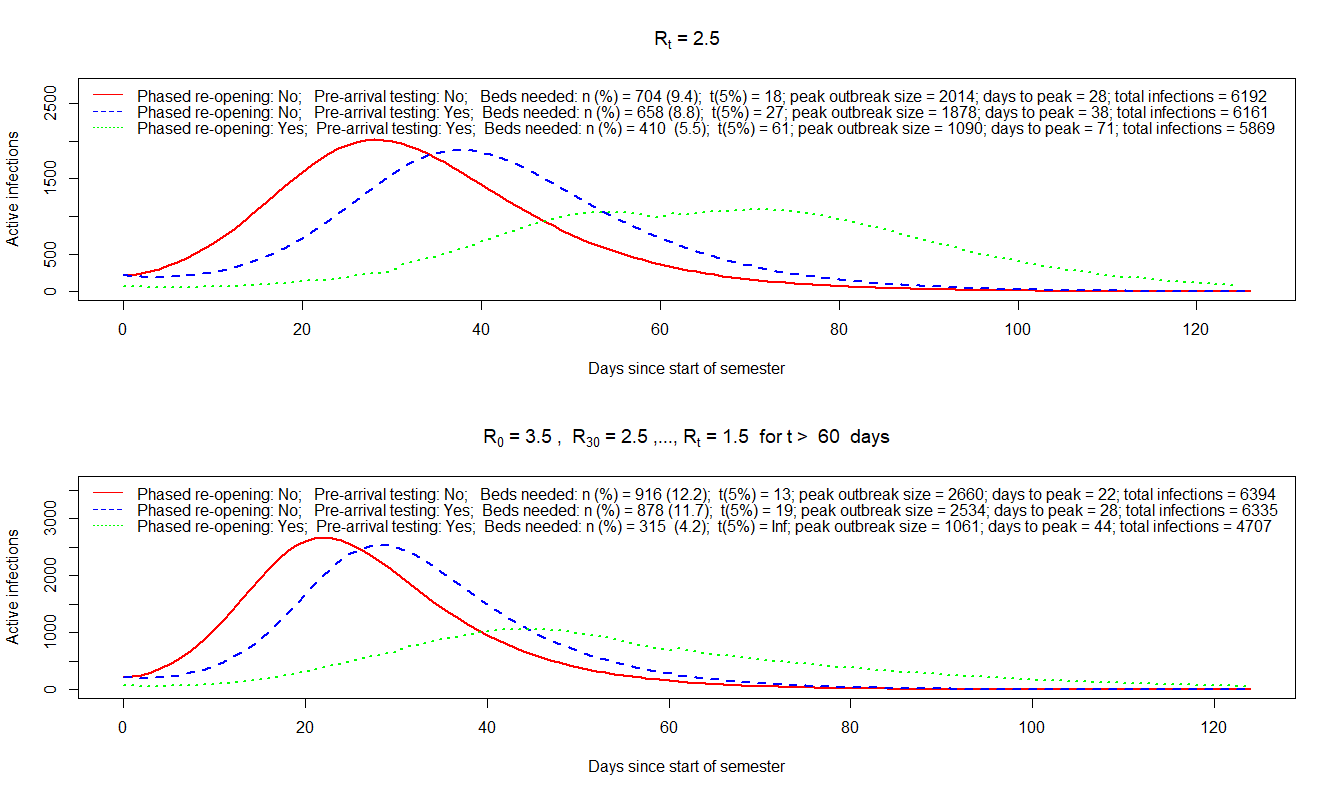


**Supplementary Figure 2.** Decreasing pre-arrival test sensitivity to 70%.


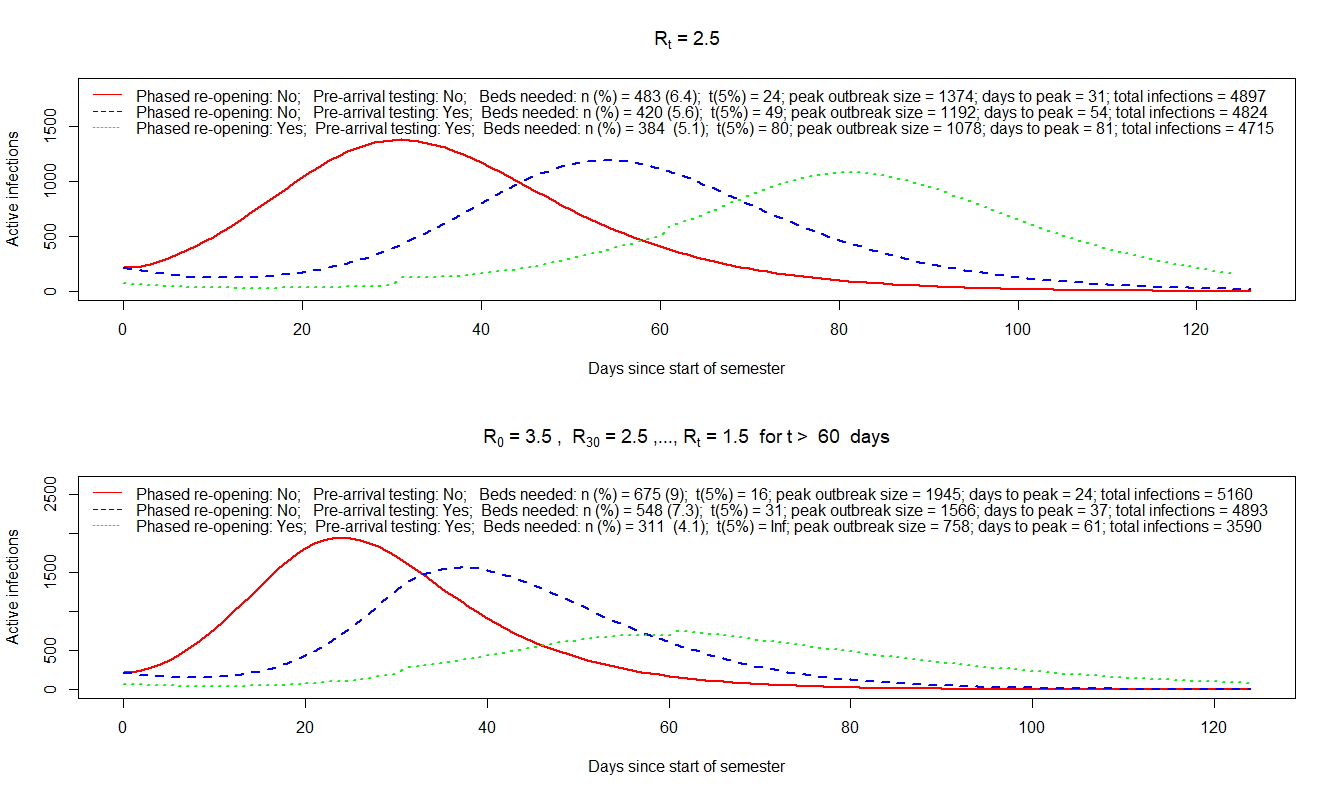


**Supplementary Figure 3.** Increasing proportion of immune individuals at the semester start to 25%.


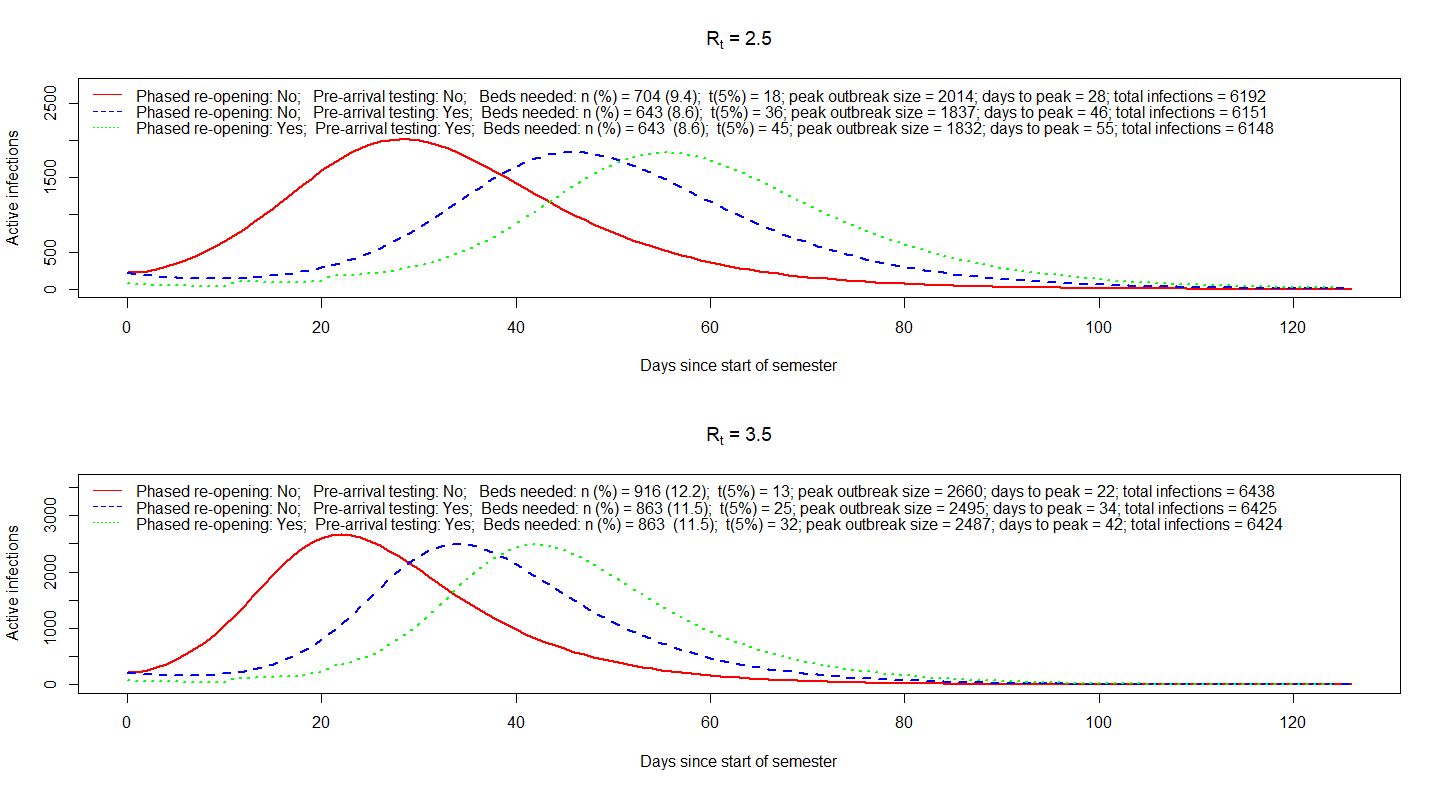


**Supplementary Figure 4.** Decreasing time between phases to 10 days.


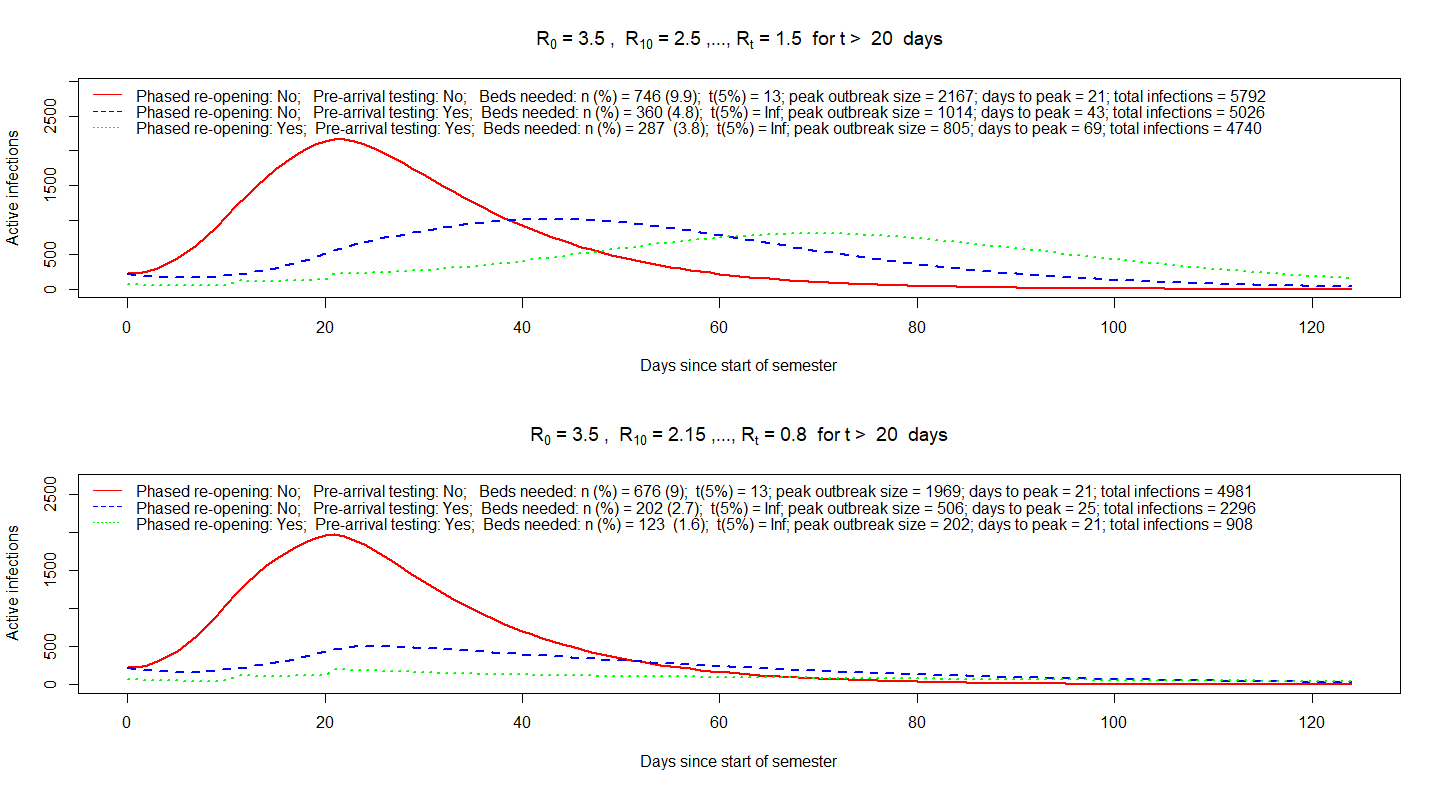


**Supplementary Figure 5.** Improving *R_t_* under settings in Supplementary Figure 4.
